# Homœopathic Prescribing

**Published:** 1889-01

**Authors:** Hayes C. French

**Affiliations:** San Francisco


### HOMŒOPATHIC PRESCRIBING.

HAYES C. FRENCH, M. D., SAN FRANCISCO.

The November number of the PHYSICIAN was as usual replete with good things, and the discussion of Dr. Gee's paper upon "Peculiar Symptoms" touched practical and vital points on the question, not only of the successful selection of a remedy, but also the ability to hold the confidence of patients and their influential friends while that remedy is being applied. Having been won to Homœopathy from a life-long antipathy to its tenets, my own struggle into faith gives me some authoritative conception of the honest doubts with which the new convert scans the first approach of the priests of Hahnemann. It must be remembered that the multiplying thousands of homœopathic physicians in the land, means a rapid and constant conversion of the people from the old to the new method of practice, and the question of the advisability of the physician making a peripatetic library of himself, may have a vital bearing upon the confidence of an inquiring convert, to whose mind the seeming dependence upon his books may suggest a lack of practical familiarity of his new doctor with the doctrines he advocates. The world is suspicious of medical students, and lavishes its fondest trust upon fossilized antiquarians, to whose hoary craniums it credits all book-lore, though they be in fact but the cephalic grave of scantest memories.

Dr. Hitchcock's modest estimate of his knowledge of materia medica, while betraying a refreshing candor, we will venture to pledge, can be refuted out of his own mind and mouth, and that he could readily give the characteristics, not only of "a dozen remedies," but of a dozen dozen, if put to the test. Without his confidence we have no patient, and frequently that confidence will depend upon the doctor's ability to make an off-hand prescription. The best homœopath we ever knew, failed utterly for want of tact in the matter of using books at the bedside. The successful settlement of this question demands a keen discrimination, and if this be used, the man who to-day would attribute your devotion to your tomes to ignorance, will to-morrow, in the light of his new-found faith, boast that *his* doctor, like his lawyer, studies his case before venturing a remedy; hence, we cannot see any hope of true success as prescribers without a general and constantly growing knowledge of the leading characteristics of our principal remedies. If the call is urgent, we must have an instantaneous grasp of its salient needs, and act with correspondent promptness. Afterward we can obtain the best possible details of the symptomatology, and retire to meditation and the books, and we have no doubt but that the most rigid adherents of the book method would much less frequently, than in their modesty they now suppose, be compelled to change the remedy thus selected.

There is a sort of unconscious cerebration accompanying the process of acquiring materia medica, which impresses facts upon the mind, not always in the scope of language to express, but which give their possessor unconsciously, a physiognomy (so to say) of the remedy in relation to the disease to which it is adapted, and many a successful prescriber follows what he has fondly denominated his "intuitions," which are nothing more than the stored-up fruits of legitimate mental labor. Now does not slavish adherence to text-books, on all occasions, and in all places, have a tendency to obtund this inspirational gift?

